# Supplementary material for: Mettl5 coordinates protein production and degradation of PERIOD to regulate sleep in Drosophila
Source: eLife. 2026 May 8;14:RP103427. doi: 10.7554/eLife.103427 (PMC13155753; doi:10.7554/eLife.103427)
Supplement: Figure 4—source data 1. [file elife-103427-fig4-data1.zip › Figure 4-Source Data 1/Figure 4—Source Data 1.pdf]

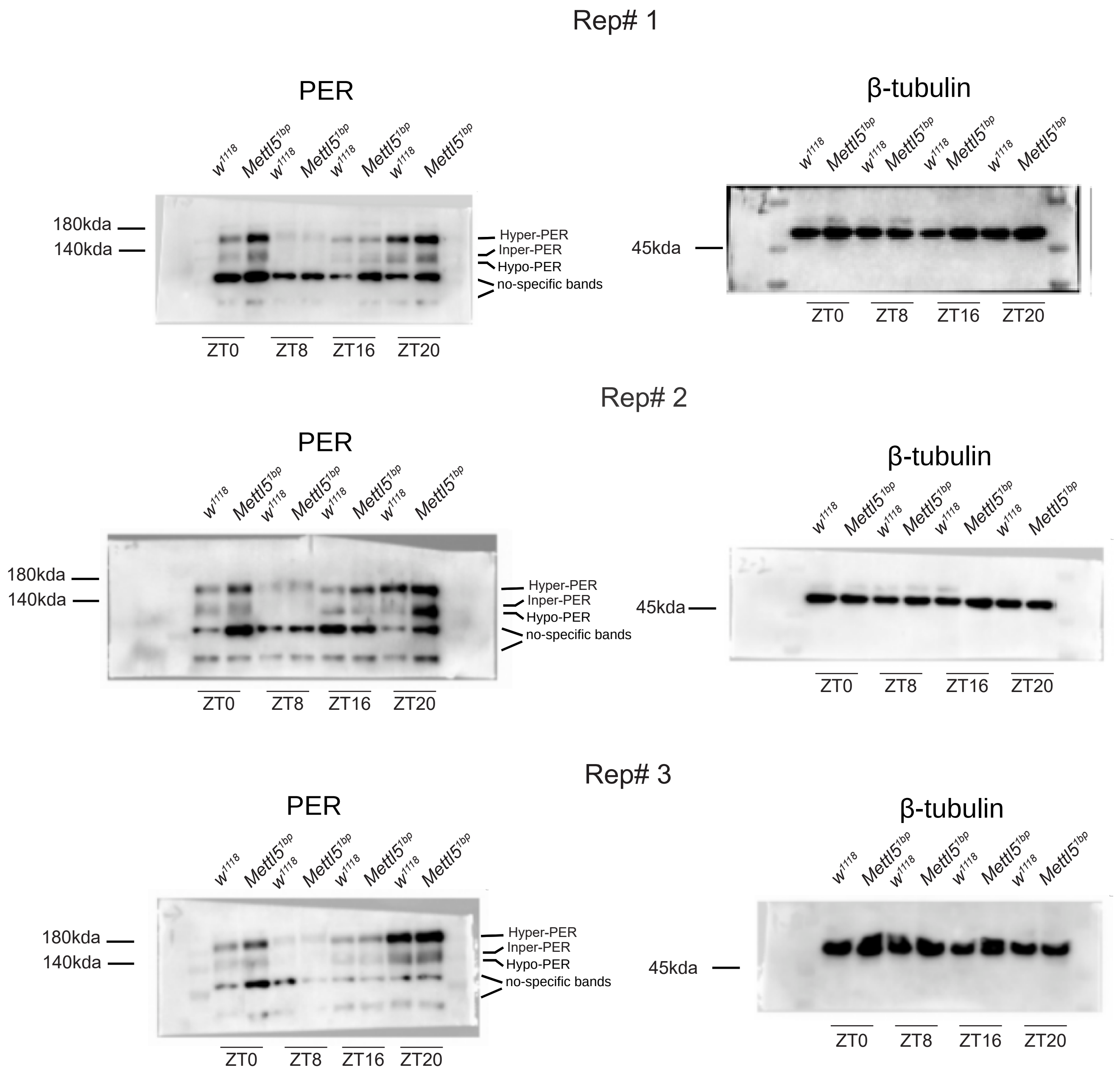

Figure 4—Source Data 1. Original membranes for PER and β-tubulin western blots shown in Figure 4E.Original scans of the full membranes from three independent biological replicates (Rep# 1, Rep# 2, and Rep# 3) are provided. Biological replicate #1 was used as the representative blot in Figure 4E. For each replicate, the PER blot is displayed on the left, and the corresponding β-tubulin blot (loading control) is shown to its right. Protein molecular weight markers (kda) are indicated on the left of each membrane. On the PER blots, brackets on the right indicate the different phosphorylation states of PER (Hyper, Inper, and Hypo), as well as lower molecular weight non-specific bands that were excluded from quantification. Samples were collected from w^{1118} and *Mettl5*^{1bp}/+ fly heads at four Zeitgeber time points (ZT0, ZT8, ZT16, and ZT20).
