# Supplementary figures and images for: Mettl5 coordinates protein production and degradation of PERIOD to regulate sleep in Drosophila
Source: eLife. 2026 May 8;14:RP103427. doi: 10.7554/eLife.103427 (PMC13155753; doi:10.7554/eLife.103427)

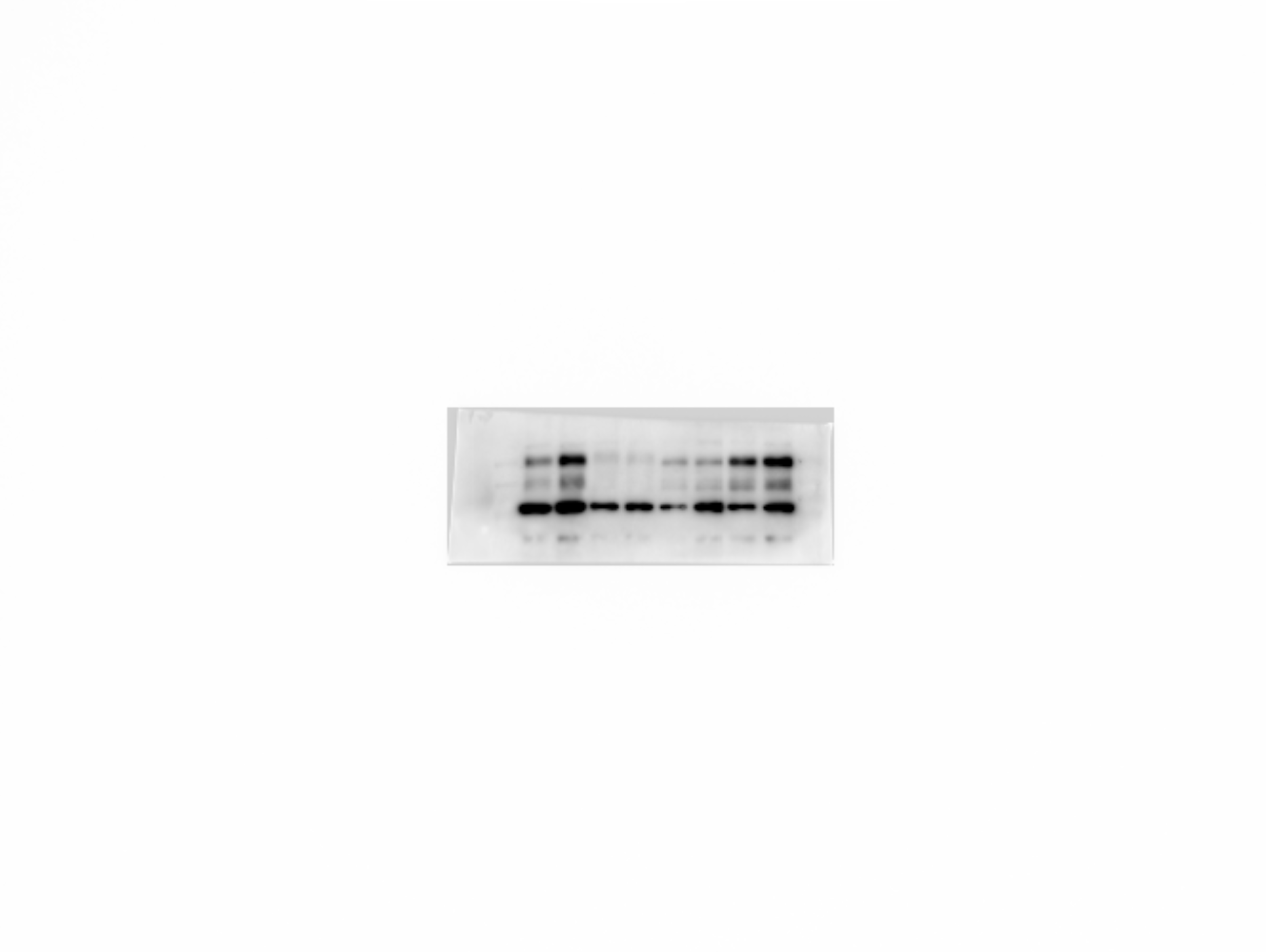

Supplement: Figure 4—source data 2. [file elife-103427-fig4-data2.zip › Figure 4-Source Data 2/PER repeat1.tif]

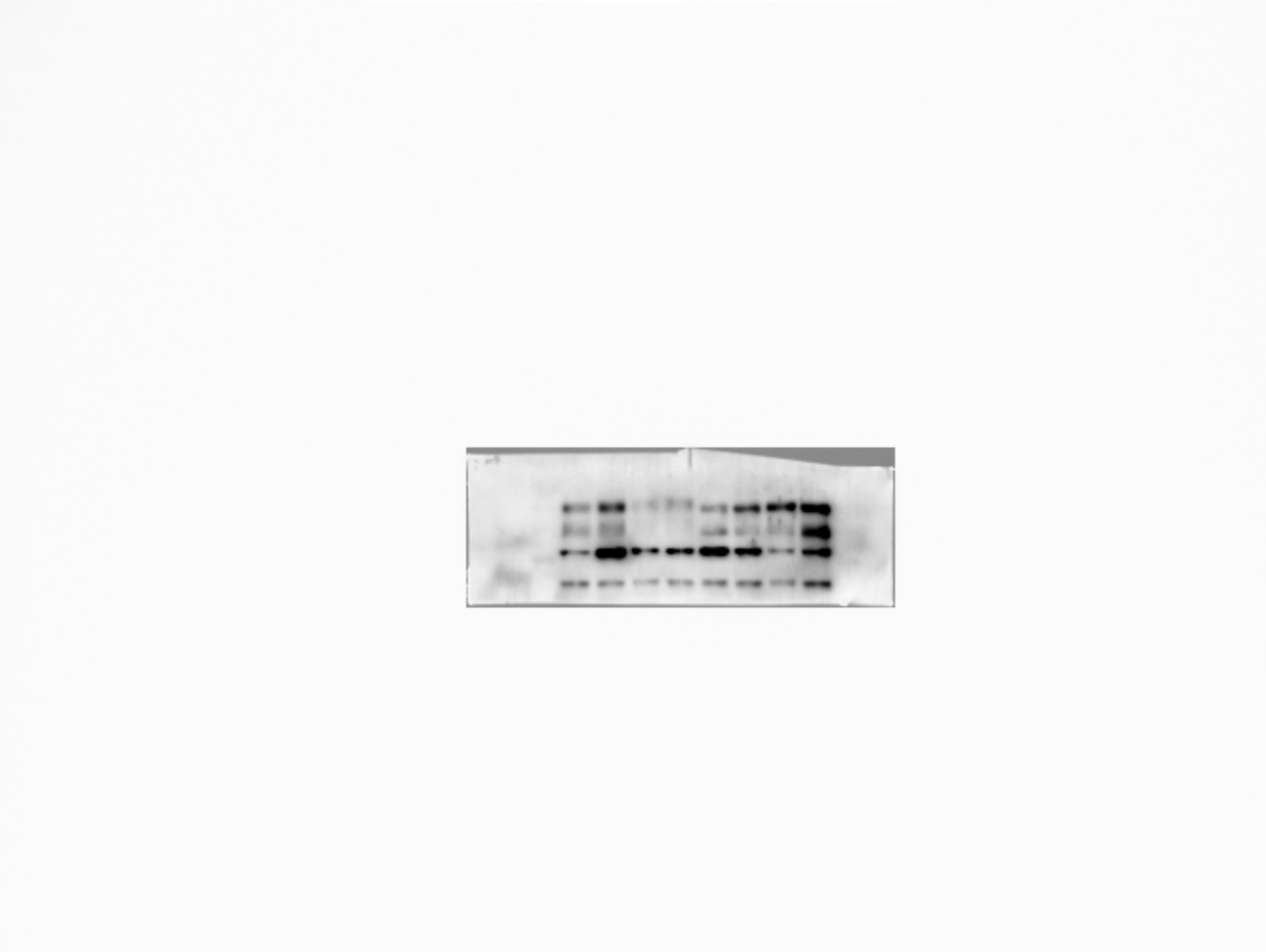

Supplement: Figure 4—source data 2. [file elife-103427-fig4-data2.zip › Figure 4-Source Data 2/PER repeat2.tif]

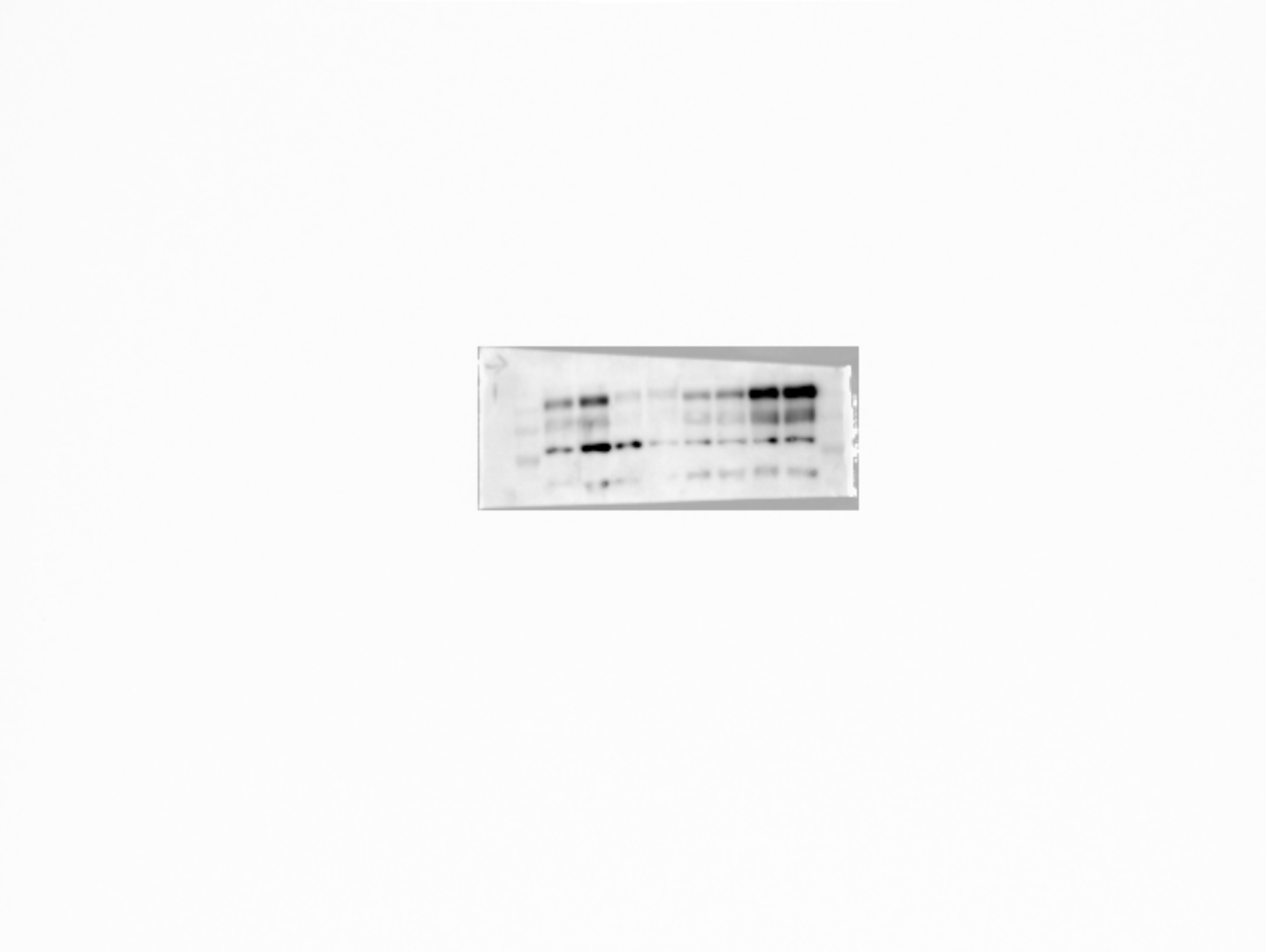

Supplement: Figure 4—source data 2. [file elife-103427-fig4-data2.zip › Figure 4-Source Data 2/PER repeat3.tif]

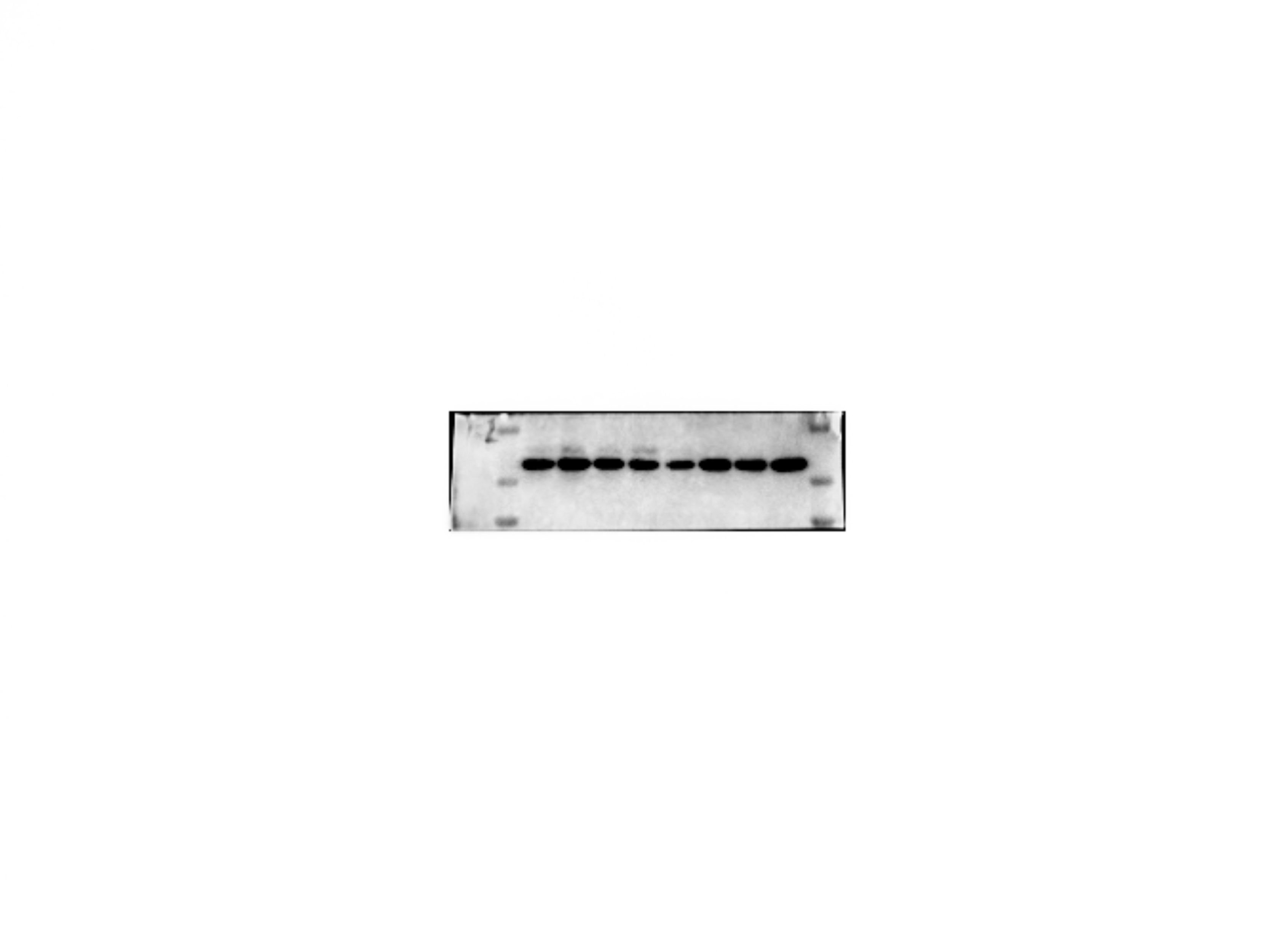

Supplement: Figure 4—source data 2. [file elife-103427-fig4-data2.zip › Figure 4-Source Data 2/β-tubulin repeat1.tif]

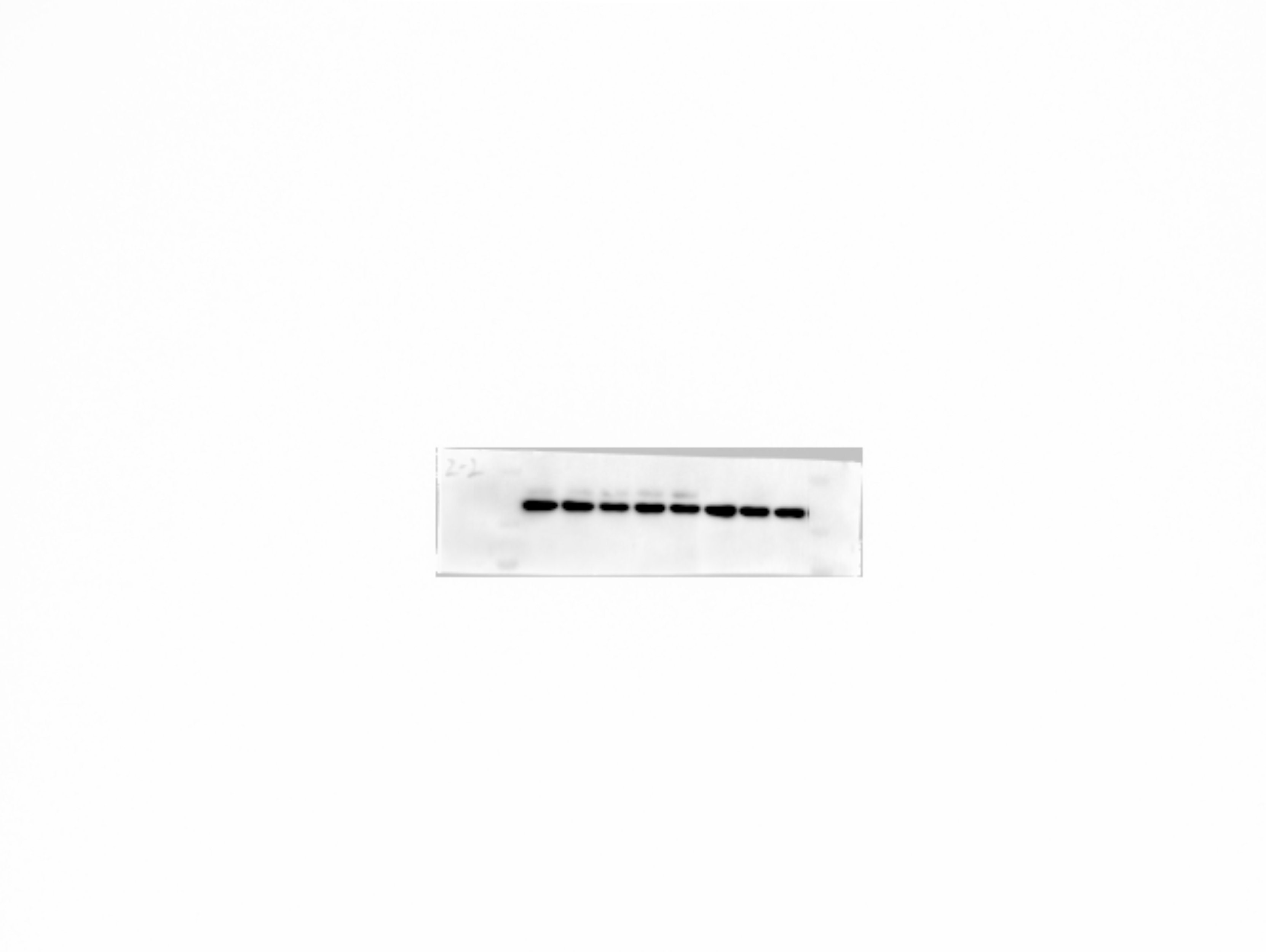

Supplement: Figure 4—source data 2. [file elife-103427-fig4-data2.zip › Figure 4-Source Data 2/β-tubulin repeat2.tif]

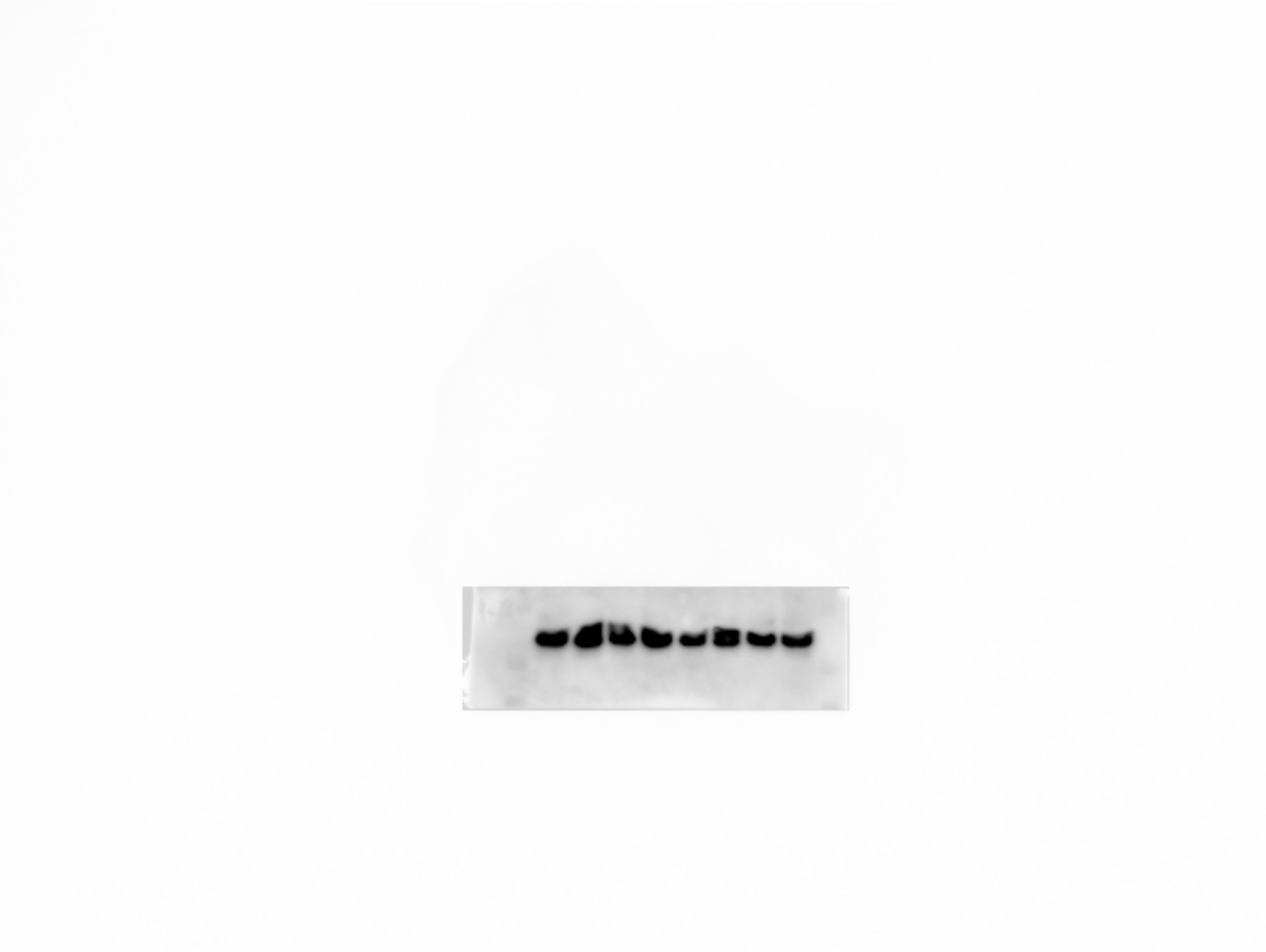

Supplement: Figure 4—source data 2. [file elife-103427-fig4-data2.zip › Figure 4-Source Data 2/β-tubulin repeat3.tif]
